# Electric Field Assisted Femtosecond Laser Preparation of Au@TiO_2_ Composites with Controlled Morphology and Crystallinity for Photocatalytic Degradation

**DOI:** 10.3390/ma14143816

**Published:** 2021-07-08

**Authors:** Xiaojie Li, Xin Li, Pei Zuo, Xiaozhe Chen, Misheng Liang, Le Ma

**Affiliations:** Laser Micro Nanofabricat Lab, Beijing Institute of Technology, Beijing 100081, China; xjlj_08@163.com (X.L.); zuopei1990@163.com (P.Z.); tlz870240502@163.com (X.C.); 3120170227@bit.edu.cn (M.L.); 3120185107@bit.edu.cn (L.M.)

**Keywords:** electric-field-assisted, femtosecond laser, Au@TiO_2_ composites, photocatalytic degradation

## Abstract

TiO_2_ is popular in photocatalytic degradation dye pollutants due to its abundance and its stability under photochemical conditions. Au loaded TiO_2_ can achieve efficient absorption of visible light and deal with the problem of low conversion efficiency for solar energy of TiO_2_. This work presents a new strategy to prepare Au nanoparticles-loaded TiO_2_ composites through electric−field−assisted temporally−shaped femtosecond laser liquid-phase ablation of Au^3+^ and amorphous TiO_2_. By adjusting the laser pulse delay and electric field parameters, gold nanoparticles with different structures can be obtained, such as nanospheres, nanoclusters, and nanostars (AuNSs). AuNSs can promote the local crystallization of amorphous TiO_2_ in the preparation process and higher free electron density can also be excited to work together with the mixed crystalline phase, hindering the recombination between carriers and holes to achieve efficient photocatalytic degradation. The methylene blue can be effectively degraded by 86% within 30 min, and much higher than the 10% of Au nanoparticles loaded amorphous TiO_2_. Moreover, the present study reveals the crystallization process and control methods for preparing nanoparticles by laser liquid ablation, providing a green and effective new method for the preparation of high-efficiency photocatalytic materials.

## 1. Introduction

Dyes are a major class of synthetic organic compounds used in various areas of industries (textiles, pharmaceuticals, rubber, etc.). Nearly 50,000 tons of dyes are released into the environment each year, requiring the removal or degradation of hazardous substances. Meanwhile, dye contaminants from groundwater and surface water remain a huge global challenge [1]. The reduction of these dye compounds from industrial wastewater has been achieved using chemical, physical, and biological methods. However, these methods are time consuming and expensive, even leading to chemical disposal problems [2,3,4]. TiO_2_ is considered the most common photo catalyst due to its cheapness, abundance, non−toxicity, and stability under photochemical conditions with wide application in the last decades [5,6]. Unfortunately, TiO_2_ has a bandgap of 3.2 eV, which only absorbs the ultraviolet portion of sunlight (UV, less than 5% of the total solar spectrum energy) [7], determining the limit of its capability of the conversion efficiency for solar energy [8,9]. As a result, the efficient absorption and conversion of sunlight by tuning the band gap of TiO_2_ has become the main research direction of TiO_2_ catalysts [10,11]. Among them, TiO_2_ loaded with noble metal nanoparticles (NPs) has received a lot of attention, because the hot electrons generated by the light-excited plasma of noble metal NPs can cross the Schottky barrier between the Fermi level of noble metal and the conduction band of semiconductor (TiO_2_) [12], thus achieving efficient absorption of visible light [13,14,15]. The construction of the interface between noble metal and TiO_2_ is of particular importance because the excitation of noble metal particles by light generates electromagnetic fields that will also enhance the excitation efficiency of TiO_2_. Recently, surfactants were commonly used to make Au nanostructures which are adsorbed on the surface of semiconductors [16,17]. However, these surfactants would create a barrier between Au and TiO_2_, hindering electron transfer [18,19]. In most cases, calcination removes the surfactant, but for non-spherical AuNPs (e.g., nanostars), hot electrons are generated to enhance the excitation efficiency of TiO_2_ by localized surface plasma resonance (LSPR) excited by light near the metal-semiconductor interface, while high temperature causes nanoparticles to remodel in the high curvature region [20], causing in the loss of the LSPR mode in the near infrared and affecting their photocatalytic activity [21,22,23]. As a result, the construction of composite materials of non-spherical AuNPs and TiO_2_ without surfactants and avoiding the destruction of the non-spherical nanoparticle morphology is an important direction to effectively improve the photocatalytic activity. Nevertheless, it has also been an obstacle that has not been overcome.

As a green, safe and controllable processing tool, ultrafast laser liquid-phase ablution is one of the most important tools for nanomaterial preparation [24,25]. The laser-matter interaction produces nanomaterials with remarkable tenability, such as size distribution, agglomeration state/dispersion, crystal structure, surface area and porosity, surface charge, shape/morphology, and solubility/solubility [26,27,28]. In this work, we propose the one-step reduction growth of AuNPs on amorphous TiO_2_ surface by electric-field-assisted temporally-shaped femtosecond(fs) laser liquid-phase ablation (ETLAL) of TiO_2_ and HAuCl_4_ suspensions; non-spherical AuNPs with anisotropy, such as Au Nano spheres, Au nanoclusters (AuNCs), and Au nanostars (AuNSs), are controllably prepared by applying additional currents to influence the aggregation and condensation processes of NPs in combination with tuning the laser pulse shape. In addition, AuNPs attached to TiO_2_, depending on their morphology, will generate different degrees of LSPR when subjected to laser irradiation. At the same time, the local high temperature caused by LSPR can achieve partial crystallization of amorphous TiO_2_. AuNSs and TiO_2_ in mixed crystalline phase form composites, due to the partially crystallized TiO_2_ expanding its surface area, while significantly increasing the photochemical reaction area. The proposed method not only reveals the crystallization process and the means of regulation of NPs formed by laser liquid phase ablation, but also provides a new strategy for the construction of composite photo catalysts.

## 2. Experimental

### 2.1. Preparation of Au^3+^ and TiO_2_ Hydrate Suspensions

The Au^3+^ and TiO_2_ hydrate suspensions were prepared by employing the sol-gel method (e.g., Figure S1a). The specific synthesis method was as follows: (1) a reaction dish containing 10 mL of anhydrous ethanol was placed on a magnetic stirrer with continuous stirring; (2) 1 mL of butyl titanate (TBOT, Aladdin Biochemical Technology Co., Ltd., Shanghai, China) was dispersed as a precursor and placed on a magnetic stirrer with stirring; (3) 10 mL of distilled water was injected quickly using a syringe, (4) subsequently 1 mL of 0.1 mol L^−1^ HAuCl_4_ (Aladdin Biochemical Technology Co., Ltd., Shanghai, China) prepared in advance was dropped to completely co-hydrolyze TBOT and HAuCl_4_ in water and alcohol solvent; (5) a pale yellow suspension was created by ultra-sonication for 40 min to obtain well-dispersed Au^3+^ and amorphous TiO_2_ hydrate (TiO_2_-2H_2_O or Ti(OH)_4_) dispersion. The total reaction equation for the hydrolysis of butyl titanate in ethanol medium was [29]:

(1) $$\mathrm{Ti}{\left(O-\mathrm{Bu}\right)}_{4}+4H_{2}O\to \mathrm{Ti}{\left(\mathrm{OH}\right)}_{4}+4{\mathrm{CH}}_{3}{\mathrm{CH}}_{2}{\mathrm{CH}}_{2}{\mathrm{CH}}_{2}\mathrm{OH}\;$$

The Ti(OH)_4_ in solution was extremely unstable, which could be further dehydrated to form amorphous TiO_2_ (a-TiO_2_) at room temperature. At this point, the Au^3+^ and TiO_2_ hydrate suspensions required for the experiment were prepared. Figure S2 presents the morphology and elemental composition of Au^3+^ and TiO_2_ hydrate suspensions

### 2.2. Electric Field Assisted Laser Processing System

Temporally-shaped fs laser: Fs laser (800 nm, 35 fs, 1 kHz, Spectra Physics, Inc., Milpitas, CA, USA) pulses were shaped into double subpulses (1:1 energy ratio). The delay time between double sub-pulses could be tuned ranging from femtoseconds to 10s of picoseconds by the position of mobil mirror driven by a linear translation stage. Besides, the total energy of the laser pulse in the experiment is 0.4 uJ.

Processing time: The fs laser beam was focused onto the Au^3+^ and TiO_2_ hydrate suspension through a Plano-convex lens for 50 min.

Move method: The computer controls the horizontal movement of the six-degree-of-freedom panning stage (Physik Instrumente, Inc., 2017, M-840.5 DG, Karlsruhe, Germany) on which the cell is located to achieve relative motion of the fs laser spot and the suspension, thus ensuring that Au^3+^ and TiO_2_ hydrate are completely processed.

Applied electric field: Platinum electrodes were fixed at both ends of the reaction cell and connected to a DC power (20 V) supply to provide experimental power.

### 2.3. Characterization of Au@TiO_2_

The surface morphology and energy dispersive X-ray spectroscopy (EDX) were investigated using a Scanning Electron Microscope (SEM, FEI Quanta 200 FEG, Hillsboro, OR, USA). The High Resolution Transmission Electron Microscope (HRTEM) was performed using a JEM-2100F (JEOL, Inc., Tokyo, Japan). The X-ray diffraction analysis was performed using a parallel beam Bruker D8 Advance X-ray diffractometer (XRD) (BRUKER AXS, Inc., Karlsruhe, Germany). Raman spectra were obtained using a Renishaw InVia Reflex spectrometer (Renishaw, Inc., Pliezhausen, Germany) with a 532 nm light source.

### 2.4. Crystallinity for Photocatalytic Degradation

Reaction device: a Pyrex glass vessel with circulating water cooling was used as the reaction vessel. In the meanwhile, the reaction temperature was kept at 4 °C.

Light source: a 300 W xenon lamp was employed to simulate the solar spectrum, and a 410 nm filter was used to filter the UV band spectrum.

Reaction system: 50 mg of photo catalyst was directly dispersed into 50 mL of 2 × 10^−5^ mol of methylene blue, and a superfine filter was applied to filter the UV band spectrum. Every 5 min, 2 mL of the reaction solution was withdrawn for subsequent absorption spectroscopy.

## 3. Results and Discussion

### 3.1. Electric-Field-Assisted Femtosecond Laser Preparation of Au@TiO_2_ Composites

**Figure 1:**
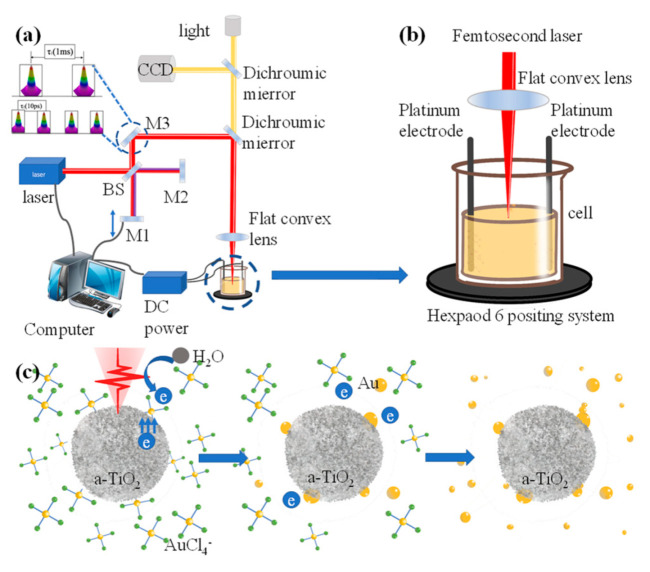
(**a**) Schematic of the optical path of the electric-field-assisted femtosecond laser liquid phase ablation; (**b**) schematic of the loading electric field; (**c**) a schematic diagram of the growth mechanism of AuNPs attached to a-TiO_2_ under the Au^3+^ and TiO_2_ hydrate suspension under femtosecond laser irradiation.

In the current experiment, Figure 1a,b shows the schematic diagram of the optical path of the electric field-assisted femtosecond laser liquid-phase ablation. Besides, the detailed illustration is provided in the experimental section. Briefly, a femtosecond laser beam was adopted and further temporally shaped into double sub-pulses with a delay ranging from femtoseconds to tens of picoseconds based moving the position of M1 (Figure 1a), and focused in the Au^3+^ and TiO_2_ hydrate suspensions.

In the present experiment, AuNPs were obtained using fs laser to ablate Au^3+^ and TiO_2_ hydrate suspensions with a low fluency, i.e., Au^3+^ reduction. The schematic diagram in Figure 1c illustrates the process of AuNPs@a-TiO_2_ NPs formation with fs laser to induce Au^3+^ reduction. The following reactions mainly occur in the experimental suspension of Au^3+^ and TiO_2_ hydrates irradiated by fs laser pulses with appropriate fluence.

(2) $${\mathrm{TiO}}_{2}\overset{\mathrm{hv}}{\to }{\mathrm{TiO}}_{2}\left(h^{+}+e^{-}\right)$$

(3) $$6H_{2}O\overset{\mathrm{hv}}{\to }2H_{2}O_{2}+4H_{2}↑+O_{2}↑$$

Meanwhile ${\left[AuCl_{4}\right]}^{-}+3e^{-}=Au^{0}+4Cl^{-}$ The basic reaction principle of Au^3+^ strong pulse laser reduction presented at this time the total reaction equation is:

(4) $${\mathrm{TiO}}_{2}\left(h^{+}+e^{-}\right)+2{\mathrm{HAuCl}}_{4}+4H_{2}O\overset{\mathrm{hv}}{\to }2{\mathrm{Au}}^{0}+{\mathrm{TiO}}_{2}+8\mathrm{HCl}+2O_{2}↑+H_{2}↑$$

Under the excitation of fs laser, the electrons in the valence band of TiO_2_ absorb the photons, which are excited to the conduction band. The free electrons are rapidly transferred to the TiO_2_ particle surface and react with the Au^3+^ ions adsorbed in TiO_2_. Then, the Au^3+^ receives electrons to be reduced to Au atoms. Since the electron leap from valence band to conduction band is extremely rapid, the Au^3+^ attached or embedded in the surface of TiO_2_ is preferentially reduced. Partially field-driven electrons can be emitted from the surface of resonant AuNPs under femtosecond laser irradiation [30]. At the same time, the free [AuCl_4_]^−^ ions around it get electronically reduced from the formed Au^0^ and condensed into the atomic nanoclusters. After laser irradiation, the AuNPs deposited on the surface of TiO_2_ were cooled down by heat exchange and heat conduction with the solution [31], thus forming a thermal plasma effect, while the free Au atomic clusters around it, started to condense and grow on the AuNPs.

### 3.2. Morphology Characterization of Au @TiO_2_ Composite

**Figure 2:**
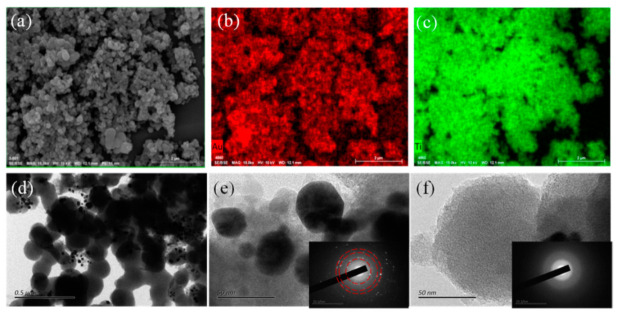
(**a**) SEM image of the sample after 30 min of Au^3+^ and TiO_2_ hydrate suspension irradiated by femtosecond laser; (**b**) EDS distribution of Au in AuNPs@a-TiO_2_ nanoparticles; (**c**) EDS distribution map of Ti in AuNPs@a-TiO_2_ nanoparticles; (**d**) Transmission electron microscope (TEM) image of AuNPs@a-TiO_2_; (**e**,**f**) The local high-resolution transmission electron microscopy (HRTEM) images in (**d**), and the insets are electron diffraction images.

The SEM image characterizes the morphology of Au@a-TiO_2_ nanoparticles obtained from Au^3+^ and TiO_2_ hydrate suspensions irradiated by femtosecond laser pulses. A representative SEM image of nanoparticles is displayed in Figure 2a. The morphology of TiO_2_ nanoparticles is not significantly different from that before irradiation. Based on the EDS diagram, it can be observed that in Au@a-TiO_2_, the Au element coincident with Ti element (Figure 2b,c), indicating that Au is evenly distributed around TiO_2_. TEM images were employed to identify the crystallinity of AuNPs obtained from Au^3+^ and TiO_2_ hydrate suspensions irradiated by fs laser pulses. TEM images of NPs (shown in Figure 2d) show that AuNPs are mostly attached to the TiO_2_ periphery, a result consistent with the mechanism of preferential nucleation and condensation of AuNPs on the TiO_2_ surface in the previous section. Figure 2e reveals the completely disordered structure of TiO_2_ NPs and the crystal structure of the decorated Au nanoclusters under such condition. The morphology of the reduced AuNPs is mainly distributed around 30 nm, and the crystallinity is high (as shown in the inset electron diffraction); the morphology of TiO_2_ after fs laser pulse ablation is shown in Figure 2f, and the electron diffraction pattern of TiO_2_ after laser ablation treatment has no obvious diffraction (as in the inset), confirming the amorphous state of TiO_2_ NPs. It can be assumed that the fs laser liquid phase ablation does not change the crystalline state of TiO_2_ in this stage.

Fs laser liquid-phase ablation has to achieve ionization of water, excitation of surface electrons of TiO_2_, as well as local electronic heating of AuNPs during the intermediate process of their crystallization. As a result, multi-pulse ultrashort laser irradiation would be the method which can be used to improve the processing efficiency. According to the nucleation and growth mechanism of nanocrystals, in addition to Ostwald’s ripening theory [32], Penn and Banfield also proposed some crystal growth modes such as directed attachment and directed aggregation [33]. In the crystallization pathway, large particles were grown from small primary particles by directed attachment, in which neighboring NPs self-assembled by sharing a common crystallographic orientation and combining these particles at a planar interface to lower the overall energy of the system [34,35]. Then, we attempted to vary the processing environment (applying an additional electric-field to change the additive light parameters) at the same laser fluence to investigate the effect of the electric field as well as the nature of the laser pulse on the Au crystal growth process.

After fs laser irradiation of Au^3+^ and TiO_2_ hydrate suspensions, AuNPs formation could be observed in all methods. Figure S3a–c shows the AuNPs prepared under fs laser conditions, mainly spherical structure with diameter distribution around 30 nm while the distribution is very obviously inhomogeneous. Figure S3c shows the HRTEM image of AuNPs prepared under this method, clearly demonstrating the single crystal lattice stripe of AuNPs with good crystallinity. The AuNPs obtained after the use of temporally-shaped fs laser ablation Au^3+^ and TiO_2_ hydrate suspension is still a spherical structure, and the morphology is similar to the previous method. Nevertheless, narrower distribution and more uniform particle size of AuNPs can be obtained by irradiating with a temporal fs laser. It is due to the more uniform energy distribution of the temporally-shaped fs laser than the conventional fs laser. Meanwhile, as presented in Figure S3d–f, the AuNPs polycrystalline ratio prepared under this processing condition is higher. According to the previously described nucleation and growth mechanism of AuNPs in TiO_2_ hydrate suspension, multiple pulses are more favorable for TiO_2_ to release electrons and thus Au^3+^ can absorb electrons for reduction. Therefore, the AuNPs here are condensed by more and finer clusters of Au atoms, The AuNPs here are formed by the condensation of more and smaller clusters of Au atoms. However, after the individual Au clusters are re-condensed, the energy provided by the laser is not sufficient to re-melt and re-condense them. Consequently, the original lattice structure is still retained and the AuNPs in Figure S3f has a polycrystalline structure.

**Figure 3:**
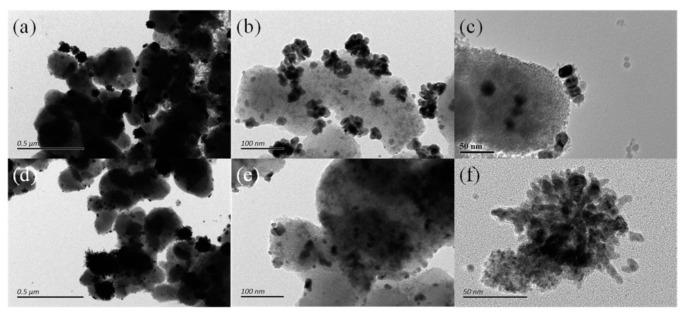
Electric field-assisted fs laser liquid phase ablation to prepare Au@TiO_2_ composite materials. (**a**–**c**) TEM images of AuNCs@TiO_2_ prepared by electric field-assisted fs laser; (**d**–**f**) TEM images of AuNSs@TiO_2_ prepared by electric field-assisted temporally-shaped fs laser.

The structure of the generated AuNPs changed significantly after the introduction of the electric field. Based on conventional fs laser pulses, the electric field was applied at both ends of the Au^3+^ and TiO_2_ hydrate suspensions. The prepared AuNPs was no longer a spherical structure, but a distinct Au nanocluster structure (Au nano-cluster, AuNCs, also as raspberry-like Au nanostructures), and the AuNCs particle size increases significantly, with an average diameter of approximately 50 nm (Figure 3a,b). During the crystallization process, the asymmetric structure is mainly due to the directional guiding effect of the applied electric field on the clusters of Au atoms to be aggregated and condensed. The HRTEM images demonstrated that AuNPs are structured by smaller Au Nano blocks that are composed of a large number of subunits with sub-nano blocks connected to each other (Figure 3c). Moreover, the corresponding HRTEM analysis shows that in Figure 3a,b the AuNCs are connected by a large number of small NPs through a common crystallographic orientation. Therefore, it is suggested that this structure formation may consist of three steps, the first step being the formation of Au atomic clusters derived from the quenching of laser-induced plasma at the liquid–plasma interface, and the second step being the formation of bulk AuNPs aggregated from primary nanocrystals by an oriented attachment mechanism. Finally, these Au atomic cluster nanocrystals formed in the first step and the bulk AuNPs formed in the second step function as building blocks to form AuNCs by directional attachment.

**Figure 4:**
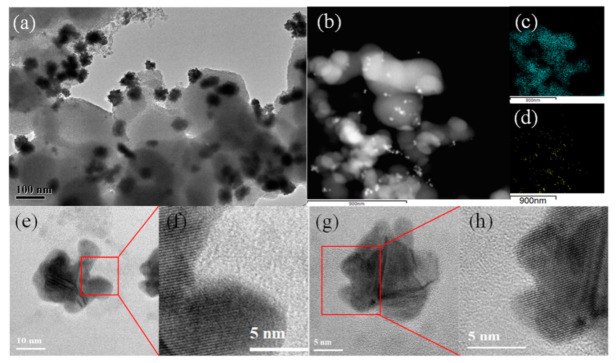
(**a**) The TEM images of AuNCs@a-TiO_2_ prepared via electric-field-assisted femtosecond laser liquid-phase ablation the hydrate suspensions of Au^3+^ and TiO_2_, (**b**,**c**) are the HRTEM images; (**d**) The TEM images of AuNSs @a-TiO_2_ prepared via electric-field-assisted temporally-shaped femtosecond laser liquid-phase ablation the hydrate suspensions of Au^3+^ and TiO_2_; (**e**,**g**) The HRTEM images of AuNSs; (**f**,**h**) are the local FFT transformed images of the corresponding regions of (**e**,**g**).

Additionally, the size distribution of the AuNPs prepared by ETLAL of Au^3+^ and TiO_2_ hydrate suspensions was similar to that of AuNCs (Figure 3d–f). However, the morphology of AuNPs has more obvious anisotropy, and the nanostar (nano-star) structure can be clearly observed in the HRTEM image (Figure 3f). Thus, it can be concluded that the AuNSs’ formation is mainly influenced by both the laser pulse morphology and the applied electric field. Due to the energy distribution, etc., the temporally-shaped fs laser makes it easier to produce more tiny Au clusters at the early stage of Au^3+^ reduction. When the applied electric field guides these Au clusters to move and attach, there are more directions to choose. Besides, the aggregation and condensation process are similar to AuNCs’ formation. Figure 4 illustrates the wide distribution and structural characteristics of AuNSs in the composite material obtained under the EFTLAL processing method. The distribution of Ti and Au in Figure 4c,d is consistent with the distribution in HRTEM images (Figure 4a,b), where AuNSs are more evenly attached to TiO_2_. Figure 4e,g are the HRTEM images of AuNSs, and Figure 4f,h are the high-resolution imagea of the corresponding structures, that confirmed the protrusions of AuNSs have the same crystal phase. Therefore, under the EFTLAL processing method, the star-shaped nanostructure is obtained through the reduction of Au^3+^ and the evolution of the structure, rather than the aggregation of nanowires.

### 3.3. Au Nanostar Induces LSPR to Crystallize a-TiO_2_

**Figure 5:**
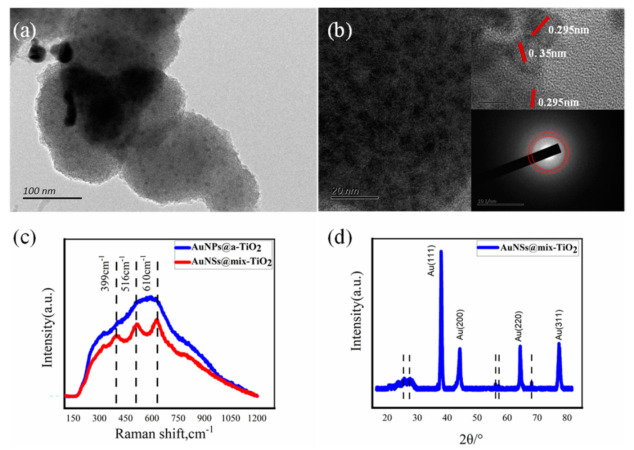
Morphology and characterization of Au nanostar and hybrid TiO_2_ composites (AuNSs@mix-TiO_2_) prepared by ETLAL. (**a**,**b**) HRTEM morphology characterization results, where the up inset in (**b**) shows the lattice spacing corresponding to the TiO_2_, and the down inset in (**b**) shows the electron diffraction pattern of TiO_2_ in the selected region; (**c**) The Raman spectrum analysis of AuNPs@a-TiO_2_ and AuNSs@mix-TiO_2_; (**d**) The XRD patterns of AuNSs@mix-TiO_2_.

As shown in Figure 5a,b, the HRTEM images clearly show the morphology of TiO_2_ in AuNSs@TiO_2_, where the lattice stripe planar spacing of the nanoscale crystalline particles is 0.351 nm and 0.295 nm, corresponding to the anatase TiO_2_ (001) and rutile phase (002), respectively [36]. This indicates that TiO_2_ partially crystallizes after AuNSs loading, constituting a mixed crystalline phase of AuNSs and TiO_2_ (AuNSs@mix-TiO_2_).

The HRTEM images and electron diffraction patterns of AuNPs@TiO_2_, which is prepared of Au^3+^ and TiO_2_ hydrate suspensions irradiated by fs laser pulses, showed that in this case TiO_2_ remains amorphous and the obtained nanocomposites are AuNPs@a-TiO_2_ (Figure S4a,b). Additionally, the same can be obtained for nanocomposites prepared under temporally-shaped fs laser for AuNPs@a-TiO_2_ materials (Figure S4c,d). However, in the nanocomposite prepared under the electric-field-assisted fs laser, an amount of crystallization TiO_2_ can be observed in the HRTEM image, while there is no obvious diffraction structure in the electron diffraction pattern. Therefore, it could be assumed that TiO_2_ undergoes mild crystallization, and the formed nanocomposite is AuNCs@mix-TiO_2_ (Figure S4e,f). Based on the comparative analysis of the Raman spectra of AuNPs@a-TiO_2_ and AuNSs@mix-TiO_2_ in Figure 5c, no obvious Raman peaks appear for AuNPs@a-TiO_2_, which is consistent with the characteristics of a-TiO_2_, while the corresponding peaks appear for AuNSs@mix-TiO_2_ at 399 cm^−1^, 516 cm^−1^, and 610 cm^−1^. This is consistent with the Raman pattern of rutile-phase TiO_2_. Besides, the appearance of these peaks can be attributed to the small amount of nano-crystalline rutile crystals in the Au@TiO_2_ product [37]. This can further confirm that there also exists crystalline rutile phase TiO_2_ present in AuNSs@mix-TiO_2_. XRD graph of the AuNSs@mix-TiO_2_ sample (Figure 5d). The diffraction peaks located at 38.2°, 44.4°, 64.4°, and 78.5°, corresponding to the (111), (200), (220), and (311) crystalline planes of Au [38], can be clearly observed, which coincide with the crystalline state of Au in the TEM image. In addition, faint diffraction peaks also appear at 27.3°, 56.4°, 58.8°, and 69.7°, and these peak positions correspond to the rutile phase of TiO_2_, also indicating the loading of AuNSs@mix-TiO_2_ with a small amount of rutile phase TiO_2_ crystals.

**Figure 6:**
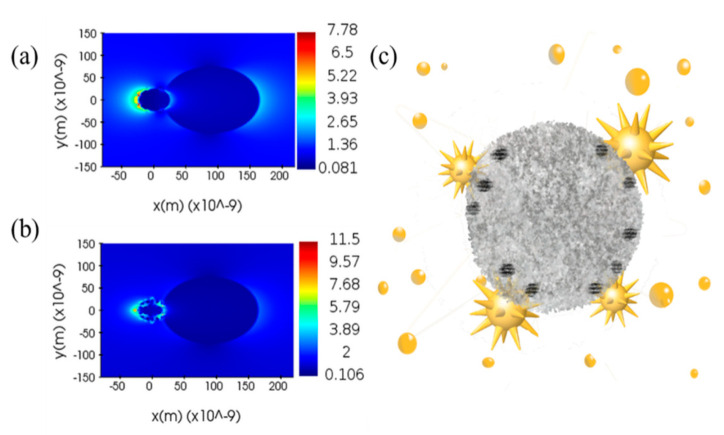
(**a**,**b**) FDTD-simulated EM field amplitude distributions around the AuNPs@TiO_2_ and AuNSs@TiO_2_, respectively; (**c**) Schematic diagram of AuNSs-induced TiO_2_ crystallization.

In the composite system of AuNPs@TiO_2_, the electric field is enhanced near the surface of NPs due to the laser excitation of AuNPs local surface plasma. Stronger near-field enhancement can be achieved if the NPs are close enough to each other. Photoionization through the surface of spherical NPs enhances the electric field by a factor of around 2–3. Nevertheless, the number density of free carriers generated by this process is in the range of 10^24^–10^25^ m^−3^, which is still not sufficient to generate a considerable effect on the substrate heating [13]. AuNPs with a radius of 45 nm is locally plasma excited by the laser at temperatures up to approximately 500 K in the vicinity [39]. The temperature of 500 K is far from the temperature requirement for a-TiO_2_ crystallization (around 800–1000 K). Therefore, AuNPs does not influence the structure of a- TiO_2_ when it is a spherical structure. However, since AuNSs has a much smaller curvature tip, the electromagnetic field enhancement around the AuNPs and AuNSs structures was analyzed with the application of the FDTD (finite-difference time-domain) method (Figure 6a,b). Meanwhile, AuNSs appears at the highest electron density at TiO_2_ and is about 1.67 times denser than the electron densest part of AuNPs. Thus, it is clear from a simple conversion of electron density to temperature that AuNSs near TiO_2_ can reach a temperature of about 900 K, completely reaching the temperature of a-TiO_2_ crystallization. In AuNSs@TiO_2_ composites, the crystallization temperature is reached at the position of AuNSs near the sharp corner of TiO_2_, and thus partial crystallization of TiO_2_ in contact with AuNSs can be achieved (Figure 6c).

### 3.4. Au@TiO_2_ Composites for Photocatalytic Degradation

**Figure 7:**
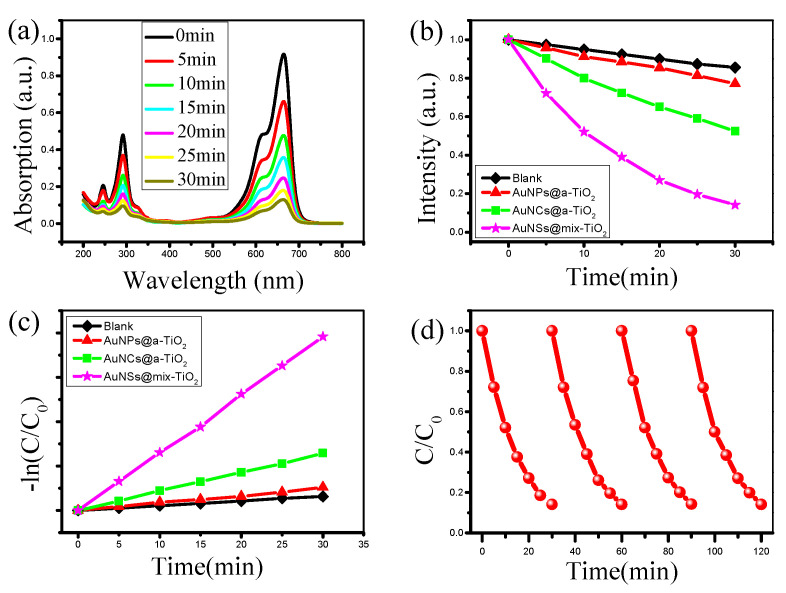
(**a**) Absorption spectrum of methylene blue solution versus 300 W xenon lamp irradiation time; (**b**) The rate of phenol degradation under visible light promoted by various AuNPs@TiO_2_ photo-catalysts; (**c**) the corresponding pseudo first-order kinetic rate plot, methyl blue (MB) concentration (C/C_0_) versus xenon lamp irradiation time; (**d**) photocatalytic stability performance of AuNSs@mix-TiO_2_.

The methylene blue molecule has a significant absorption peak near 664 nm, indicating the higher the concentration, the stronger the absorption spectrum; on the contrary, the lower the absorption spectrum. Therefore, the photocatalytic activity of the TiO_2_ catalyst can be measured by calibrating the intensity of the peak with irradiation [17,40]. Figure 7a presents the variation of the absorption spectrum of methylene blue solution in the presence of the catalyst AuNSs@mix-TiO_2_ in relation to the irradiation time. Obviously, when the irradiation time is 30 min, the intensity of the absorption spectrum of the methylene blue solution is significantly reduced, implying that the methylene blue molecules are almost completely degraded by the AuNSs@mix-TiO_2_ catalyst. To demonstrate the catalytic activity of AuNSs@mix-TiO_2_, the variation of the absorption spectrum intensity of methylene blue solution with time was compared under different catalyst conditions. Figure 7b compares the catalytic degradation rates of AuNPs@a-TiO_2_, AuNCs@mix-TiO_2_, and AuNSs@mix-TiO_2_ composites prepared by fs laser. The blank control experiment (without catalyst condition) showed that only about 10% of methylene blue molecules were degraded within 30 min, and it could be tentatively concluded that this degradation was due to the photosensitization effect. With about 86% of methylene blue molecules degraded within 30 min, the degradation rate of AuNSs@mix-TiO_2_ was significantly higher. It is demonstrated that the prepared AuNSs@mix-TiO_2_ has excellent photocatalytic degradation ability of methylene blue molecules and has great potential in alleviating environmental pollution problems. Figure 7c shows the relationship between the pseudo first-order kinetic rate constant and the length of light exposure under different conditions, where the reaction rate of AuNSs@mix-TiO_2_ is constant reaction rate with constant k = 0.06526 min^−1^, the reaction rate of AuNPs@a-TiO_2_ material is constant reaction rate constant k = 0.00529 min^−1^. Moreover, no obvious changes in the photocatalytic activity of AuNSs@TiO_2_ were observed after six photocatalytic cycles, indicating satisfactory stability in photodegrading environment pollutants (Figure 7d).

**Figure 8:**
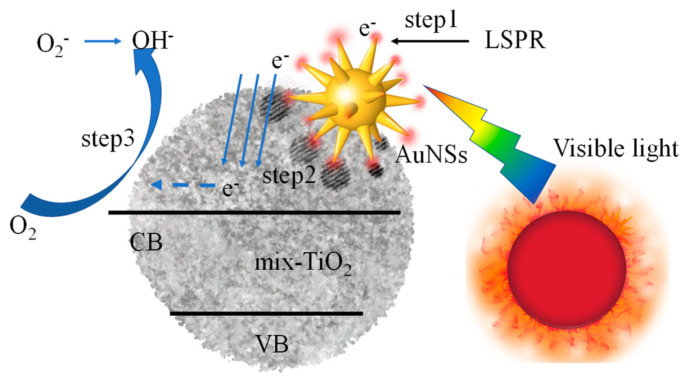
Schematic diagram of the mechanism of photocatalytic degradation of methylene blue by AuNSs@mix-TiO_2_.VB: valence band; CB: conduction band; LSPR: localized surface plasma resonance.

Based on the above results, we propose a method for the efficient degradation of azo dyes (methyl blue) using AuNSs@mix-TiO_2_ (Figure 8). The presence of AuNPs is a large electron reservoir that producing a near-field plasma on the Au-TiO_2_ interface by absorbed the visible light, and facilitates efficient charge separation by trapping light-generated holes and keeping the electrons in TiO_2_. The AuNSs would generate higher intensity LSPR [41,42], that provides more electrons for the photocatalytic degradation process. The location touched by AuNSs has been transformed from rather dense amorphous TiO_2_ to crystalline TiO_2_ with a loose structure. On the no hand, this loose structure expands the surface area of TiO_2_. As a result, the photo-generated electrons do not need to migrate to the shell surface to participate in the reduction reaction. On the other hand, the special structure facilitates the entry of reactants and the migration of products. Since most of the electrons are induced by AuNSs near the crystalline TiO_2_ surface, they can efficiently migrate to the surface without recombination, where they react with oxygen in solution, making it a strong oxidant for the efficient degradation of azo dyes.

## 4. Conclusions

AuNPs@TiO_2_ composite photo-catalysts were controllably prepared by the ETLAL method. Experiments demonstrated that the time-shaped fs laser contributes to forming smaller gold atomic clusters, while the electric field assistance can influence their motion and attachment forms. Consequently, Au nanospheres, AuNCs, and AuNSs can be controlled prepared. Among them, AuNSs@ mix-TiO_2_ composites prepared by ETLAL can enhance the photocatalytic degradation efficiency from two aspects: (1) During the synthesis of the composites, they are continuously exposed to fs laser radiation, when AuNSs attached to a-TiO_2_ as anisotropic nano-plasmas generate a large number of free electrons at the top of the structure and rapidly increase the free electrons following the increase of temperature. Additionally, it is calculated that the region where AuNSs are in contact with a-TiO_2_ at this time is a localized high temperature region. Theoretically, the temperature can reach 800–1000 K, which can completely realize the crystallization process of a-TiO_2_ that can transform a-TiO_2_ into crystalline TiO_2_. Crystalline phase mixed TiO_2_ will make the carrier motion more complicated and hinder the complex between electron-hole, thus realizing the improvement of catalytic efficiency. (2) In the photocatalytic degradation of AuNSs@mix-TiO_2_ composites, Au first absorbs visible light to excite LSPR and release a large number of free electrons. Then, photo-generated electrons are injected into O_2_ adsorbed on TiO_2_. The local work function of TiO_2_ near to interfacial oxygen adsorption site enhances the reduction of dioxygen to yield superoxide radicals. Compared with AuNPs, AuNSs could induced stronger LSPR, and injected more hot electrons into TiO_2_. This study providing a green and effective new method for the preparation of high-efficiency photocatalytic materials.

## Data Availability

Data is contained within the article or supplementary material. The data presented in this study are available in materials-14-03816.

## Supplementary Materials

The following are available online at https://www.mdpi.com/article/10.3390/ma14143816/s1 or from the author. Figure S1: Schematic of Au^3+^ and TiO_2_ hydrate suspension prepared by sol-gel method; Figure S2: (**a**) SEM image of Au^3+^ and TiO_2_ hydrate prepared by sol-gel method; (**b**) The EDS element distribution map of Au^3+^ and TiO_2_ hydrate suspension; Figure S3: (**a**) The TEM images of AuNPs @a-TiO_2_ prepared via femtosecond laser liquid-phase ablation the hydrate suspensions of Au^3+^ and TiO_2_, (**b**) and (**c**) are the HRTEM images; (**d**) The TEM images of AuNPs @a-TiO_2_ prepared via temporally-shaped femtosecond laser liquid-phase ablation the hydrate suspensions of Au^3+^ and TiO_2_; (**e**) and (**f**) are the HRTEM images, the scale bars are shown in the lower left corner of the images; Figure S4: (**a**) The TEM images of AuNPs @a-TiO_2_ prepared via femtosecond laser liquid-phase ablation; (**b**) The HRTEM images of the TiO_2_ in (**a**), the insets of (**b**) is the diffraction map of the corresponding image positions; (**c**)The TEM images of AuNPs @a-TiO_2_ prepared via temporally-shape femtosecond laser liquid-phase ablation; (**d**) The HRTEM images of the TiO_2_ in (**c**), the insets of (**c**) is the diffraction map of the corresponding image positions; (**e**)The TEM images of AuNCs @mix-TiO_2_ prepared via electric-field-assisted t femtosecond laser liquid-phase ablation; (**f**) The HRTEM images of the TiO_2_ in (**e**), the insets of (**e**) is the diffraction map of the corresponding image positions.
