# Supplementary material for: Electric Field Assisted Femtosecond Laser Preparation of Au@TiO2 Composites with Controlled Morphology and Crystallinity for Photocatalytic Degradation
Source: Materials (Basel). 2021 Jul 8;14(14):3816. doi: 10.3390/ma14143816 (PMC8303837; doi:10.3390/ma14143816)
Supplement: Supplementary file 1 [file materials-14-03816-s001.zip › materials-1269730-supplementary.pdf]

materials

MDPI

Supplementary Materials

Electric-Field-Assisted Femtosecond Laser Preparation of Au@TiO_2 Composites with Controlled Morphology and Crystallinity for Photocatalytic Degradation

Xiaojie Li, Xin Li *, Pei Zuo, Xiaozhe Chen, Misheng Liang and Le Ma

Laser Micro Nanofabricat Lab, Beijing Inst Technol, Beijing 100081, China; xjlj_08@163.com (X.L.); zuopei1990@163.com (P.Z.); tlz870240502@163.com (X.C.); 3120170227@bit.edu.cn (M.L.); 3120185107@bit.edu.cn (L.M.)

* Correspondence: lixin02@bit.edu.cn; Tel.: +86-010-68914524

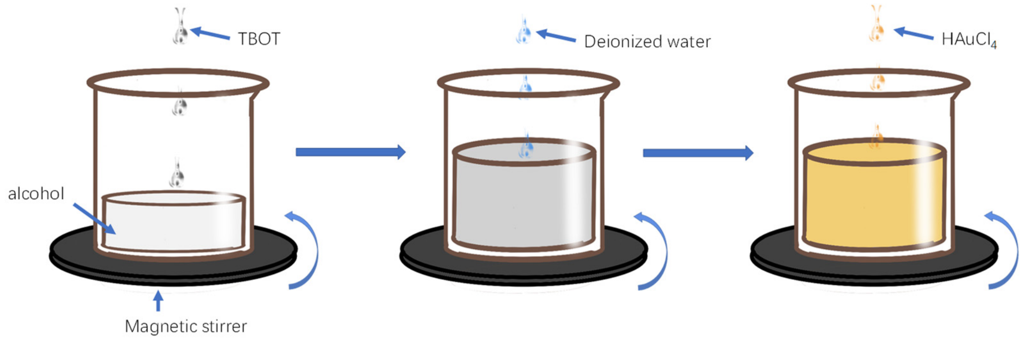

Figure S1. Schematic of Au^{3+} and TiO_2 hydrate suspension prepared by sol-gel method.

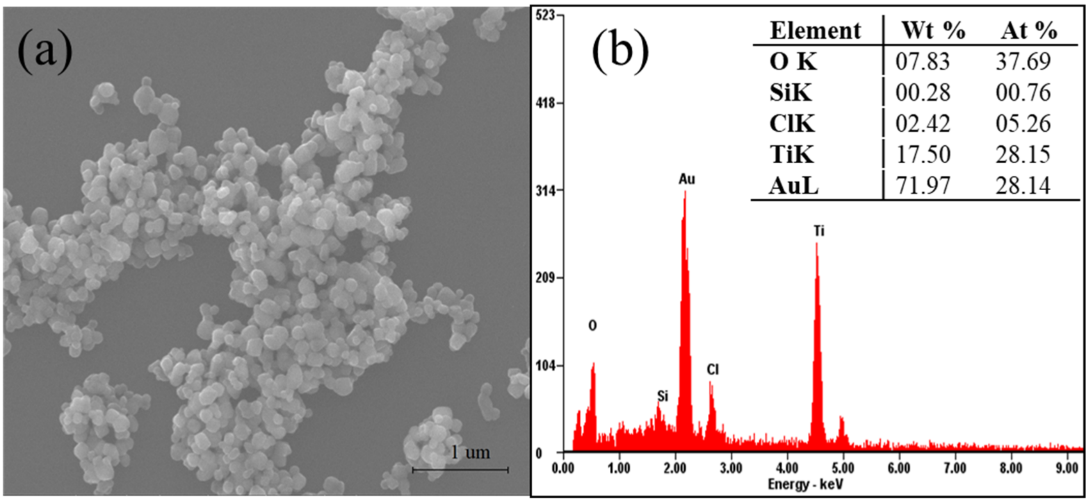

Figure S2. (**a**) SEM image of Au^{3+} and TiO_2 hydrate prepared by sol-gel method; (**b**) The EDS element distribution map of Au^{3+} and TiO_2 hydrate suspension.

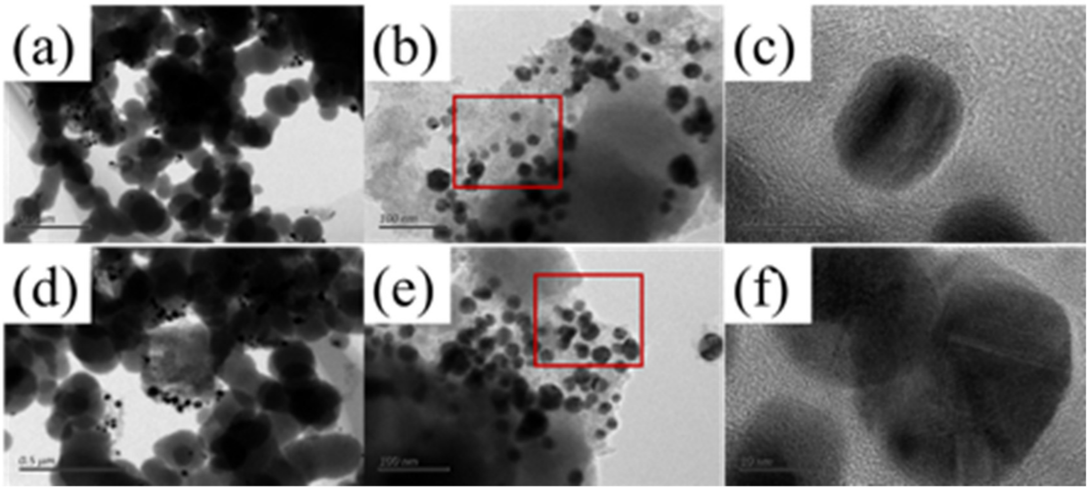

Figure S3. (**a**) The TEM images of AuNPs @a-TiO_2 prepared via femtosecond laser liquid-phase ablation the hydrate suspensions of Au^{3+} and TiO_2, (**b**) and (**c**) are the HRTEM images; (**d**) The TEM images of AuNPs @a-TiO_2 prepared via temporally-shaped femtosecond laser liquid-phase ablation the hydrate suspensions of Au^{3+} and TiO_2; (**e**) and (**f**) are the HRTEM images, the scale bars are shown in the lower left corner of the images.

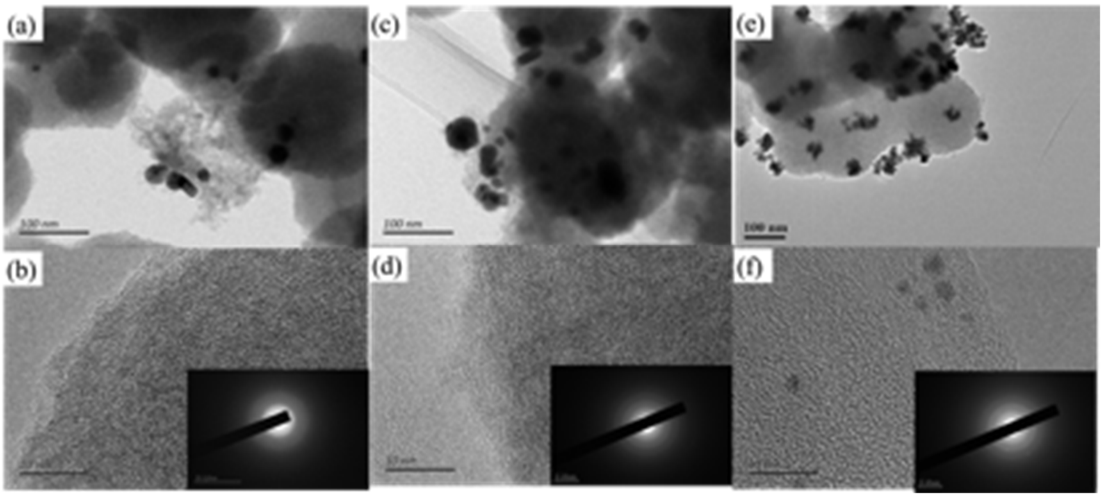

Figure S4. (**a**) The TEM images of AuNPs @a-TiO_2 prepared via femtosecond laser liquid-phase ablation ; (**b**) The HRTEM images of the TiO_2 in (**a**), the insets of (**b**) is the diffraction map of the corresponding image positions； (**c**)The TEM images of AuNPs @a-TiO_2 prepared via temporally-shape femtosecond laser liquid-phase ablation ; (**d**) The HRTEM images of the TiO_2 in (**c**), the insets of (**c**) is the diffraction map of the corresponding image positions; (**e**)The TEM images of AuNCs @mix-TiO_2 prepared via electric-field-assisted t femtosecond laser liquid-phase ablation ; (**f**) The HRTEM images of the TiO_2 in (**e**), the insets of (**e**) is the diffraction map of the corresponding image positions.
